# Spatio-temporal distribution characteristics of COVID-19 in China: a city-level modeling study

**DOI:** 10.1186/s12879-021-06515-8

**Published:** 2021-08-14

**Authors:** Qianqian Ma, Jinghong Gao, Wenjie Zhang, Linlin Wang, Mingyuan Li, Jinming Shi, Yunkai Zhai, Dongxu Sun, Lin Wang, Baozhan Chen, Shuai Jiang, Jie Zhao

**Affiliations:** 1 The First Affiliated Hospital of Zhengzhou University, Zhengzhou, Henan China; 2 National Engineering Laboratory for Internet Medical Systems and Applications, Zhengzhou, China; 3 School of Management Engineering, Zhengzhou University, Zhengzhou, China

**Keywords:** COVID-19, Visualization, Spatio-temporal distribution, Geographic hotspot, Time–space scan, China

## Abstract

**Background:**

The coronavirus disease 2019 (COVID-19) has become a pandemic. Few studies have been conducted to investigate the spatio-temporal distribution of COVID-19 on nationwide city-level in China.

**Objective:**

To analyze and visualize the spatiotemporal distribution characteristics and clustering pattern of COVID-19 cases from 362 cities of 31 provinces, municipalities and autonomous regions in mainland China.

**Methods:**

A spatiotemporal statistical analysis of COVID-19 cases was carried out by collecting the confirmed COVID-19 cases in mainland China from January 10, 2020 to October 5, 2020. Methods including statistical charts, hotspot analysis, spatial autocorrelation, and Poisson space–time scan statistic were conducted.

**Results:**

The high incidence stage of China’s COVID-19 epidemic was from January 17 to February 9, 2020 with daily increase rate greater than 7.5%. The hot spot analysis suggested that the cities including Wuhan, Huangshi, Ezhou, Xiaogan, Jingzhou, Huanggang, Xianning, and Xiantao, were the hot spots with statistical significance. Spatial autocorrelation analysis indicated a moderately correlated pattern of spatial clustering of COVID-19 cases across China in the early phase, with *Moran’s I* statistic reaching maximum value on January 31, at 0.235 (*Z* = 12.344, *P* = 0.001), but the spatial correlation gradually decreased later and showed a discrete trend to a random distribution. Considering both space and time, 19 statistically significant clusters were identified. 63.16% of the clusters occurred from January to February. Larger clusters were located in central and southern China. The most likely cluster (*RR* = 845.01, *P* < 0.01) included 6 cities in Hubei province with Wuhan as the centre. Overall, the clusters with larger coverage were in the early stage of the epidemic, while it changed to only gather in a specific city in the later period. The pattern and scope of clusters changed and reduced over time in China.

**Conclusions:**

Spatio-temporal cluster detection plays a vital role in the exploration of epidemic evolution and early warning of disease outbreaks and recurrences. This study can provide scientific reference for the allocation of medical resources and monitoring potential rebound of the COVID-19 epidemic in China.

**Supplementary Information:**

The online version contains supplementary material available at 10.1186/s12879-021-06515-8.

## Introduction

In late December 2019, an outbreak of viral pneumonia caused by an unknown aetiology was firstly reported in Wuhan City, the capital of Hubei Province in China [1]. Most patients from the initial cluster had epidemiological links to the Huanan Seafood Wholesale Market in Wuhan, where there was sale of seafood and some live animals, suggesting a possible zoonotic origin [1, 2]. However, the definitive source of the virus is still unclear. The pneumonia was later identified to be caused by a pathogen-severe acute respiratory syndrome coronavirus-2 (SARS-CoV-2), which was subsequently officially named the novel coronavirus disease 2019 (COVID-19) by the World Health Organization (WHO) [2–4]. Phylogenetic analysis revealed that SARS-CoV-2 had a positive-sense single-stranded RNA and fell within the subgenus Sarbecovirus of the genus Beta-coronavirus, with 88–89% similarity to its two closest bat-derived relatives, bat-SL-CoVZC45 and bat-SL-CoVZXC21 [2, 5–7]. However, SARS-CoV-2 is genetically distinct from SARS-CoV with about 79% similarity, and Middle East respiratory syndrome coronavirus (MERS-CoV) with only approximately 50% similarity [6–8].

Evidence of infections in family clusters and medical workers has confirmed the occurrence of human-to-human transmission [6–8]. Similar to the common flu, SARS-CoV-2 infection is mainly through contact transmission and respiratory droplets [10]. The COVID-19 outbreak spread rapidly in China and other countries. On January 30, 2020, the WHO declared that the COVID-19 epidemic was a public health emergency of international concern [11]. And WHO claimed that COVID-19 was a pandemic with an estimated death rate between 1 and 5% on March 11, 2020[11]. As of October 5, 2020, more than 35 million cases of COVID-19 have been reported in almost all countries and territories worldwide, resulting in approximately 1.04 million deaths [13]. In mainland China, until October 5, 2020, 85,482 cases of COVID-19 were officially confirmed, including 4634 deaths [14].

In terms of the ongoing global COVID-19 pandemic, a large number of epidemiological, ecological and statistical models have been employed to observe the changes, investigate the distribution, and assess the tendency of this epidemic [15, 16]. However, most of these studies focused on epidemiological and clinical characteristics, transmission indicators, and population distribution of COVID-19 [17, 18]. Relatively, studies evaluating the spatial and temporal spread of the COVID-19 pandemic are fewer, especially in developing country like China that suffered serious influence from the disease with limited medical resources [10, 19]. To date, several studies in China involving spatiotemporal distribution analysis have only discussed the temporal and spatial information of cases from the regional or provincial level, limited attentions have been paid to the nationwide city-level spatiotemporal spread of COVID-19 [10, 19, 20].

Spatiotemporal distribution analysis can reflect the temporal and spatial evolution of the COVID-19, identify significant clusters of the disease, explore the potential variation rules, and determine whether the observed space–time patterns of the epidemic are due to chance or randomly distributed [10, 12]. Besides, comprehensive spatiotemporal information of the cases is useful and imperative to further detect active and emerging clusters of COVID-19, which can inform decision makers and related stakeholders where and when to improve targeted response measures to mitigate further transmission [12, 21]. Thus, it has been suggested that conducting space–time investigation can prioritize locations for targeted prevention and control measures, rapid testing, and healthcare resources allocation [12, 22]. For these reasons, it is very important to research spatiotemporal clusters and hot spots of COVID-19 cases, especially at the city level in mainland China. This idea has been the main motivation for this research. In the present study, hotspot analysis, spatial autocorrelation, and Poisson space–time scan statistic were used to describe the spatiotemporal pattern and measure the spatial association of the COVID-19 epidemic in 362 cities in mainland China.

## Materials and methods

### Data

**Fig. 1:**
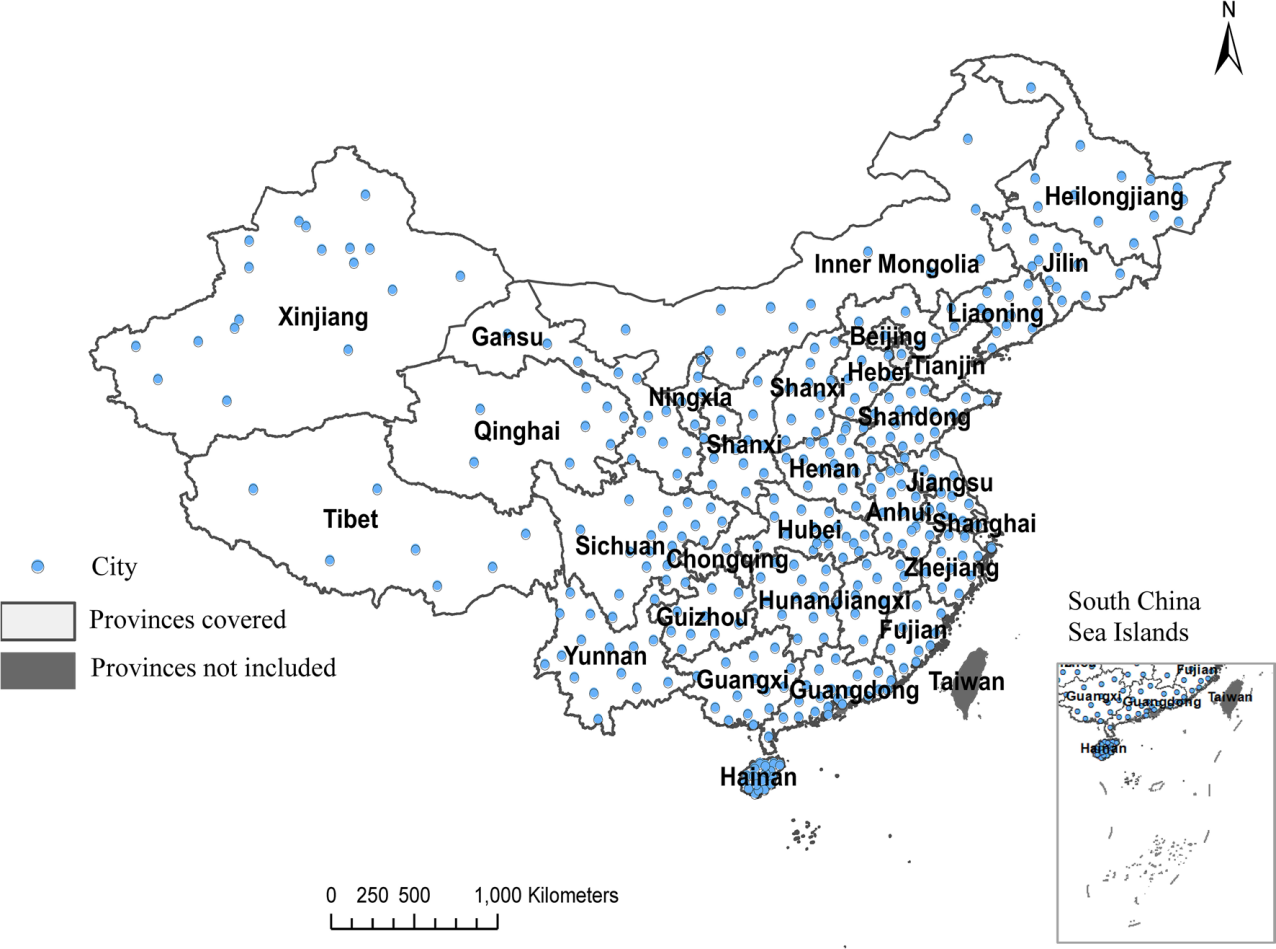
Distribution of research areas

This study belongs to spatial epidemiology, which aims to describe the spatiotemporal distribution of COVID-19 cases in the Chinese population. The COVID-19 cases and location data were collected from official websites of the National Health Commission of China and provincial Health Commissions [23]. In the present study, only the confirmed COVID-19 cases from January 10, 2020 to October 5, 2020 were considered, excluding asymptomatic infection cases. The COVID-19 cases in China were reported according to “COVID-19 Diagnosis and Treatment Plan” [24], and the diagnosed COVID-19 cases are confirmed by one of the following etiological or serological evidence: Real-time fluorescent Reverse Transcription-Polymerase Chain Reaction (RT-PCR) detection is positive for the COVID-19 nucleic acid; the results of viral gene sequencing are highly homologous with COVID-19 virus; COVID-19 IgM antibody and IgG antibody tests are positive; COVID-19 IgG antibody changes from negative to positive or the IgG antibody titer in the recovery phase is 4 times or more higher than that in the acute phase. The latter two items have been applicable since March 3, 2020*.* A total of 85,482 cases were collected from 31 provinces, municipalities and autonomous regions (Fig. 1). This study area covered 362 cities in mainland China, which included 4 municipalities, 292 prefecture-level cities, 7 districts, 30 autonomous prefectures, 3 leagues, and 26 provincial county-level cities(Additional file 1). In the spatial statistical analysis and spatio-temporal scanning analysis, 18 cases of unknown specific city information in the Xinjiang Uygur Autonomous Region were excluded. The permanent population of each city at the end of 2019 used in the time–space scanning analysis was derived from the national economic and social development statistical bulletin of each city in 2019. The geographic information, including latitude and longitude, was collected from the National Geomatics Center of China [25].

### Methods

The analysis process was divided into three steps. First, MS Excel 2013 (Microsoft Inc., Redmond, CA, USA) was used to collate the original data and explore the temporal distribution of COVID-19 cases in China by drawing statistical graphs. Second, hot spot analysis was adopted to explore the spatial distribution characteristics of COVID-19 cases in China. Finally, the global autocorrelation analysis at different time points and Poisson space–time scan statistic were applied to explore the spatiotemporal patterns of COVID-19 cases. ArcGIS Desktop software (version 10.3.1, Environmental Systems Research Institute, Inc, USA) provided tools for hot spot analysis and global autocorrelation analysis. The analysis of space–time clustering was performed using SatScan version 9.4.4 software (Kulldorff and Information Management Services Inc., Boston, MA, USA).

#### Hot spot analysis

A feature with a high value tends to attract attention, but it may not be a statistically significant hot spot. Hot spot analysis based on Getis–Ord Gi* statistics can identify statistically significant spatial clusters of high values (hot spots) and low values (cold spots) through the resultant Gi^*^ z-scores and *P*-values, which can especially be conducive to the discovery of high-incidence area of COVID-19 disease [26, 27]. Gi* values are assumed to be independently normal and identically distributed, in order to control the overall probability of Type I error for multiple tests, Bonferroni correction was used to test the significance of Gi* values. If Gi^*^ z score ≥ 3.63, it indicates that the area is a statistically significant high-value spatial cluster, that is, a disease "hot spot area"; while Gi^*^ z score ≤ -3.63 means that the area is a statistically significant low-value spatial cluster. The Getis–Ord Gi* statistic is calculated as the following formula

$${\mathrm{G}}_{i}^{*}=\frac{\sum_{j=1}^{n}{w}_{ij}{x}_{j}-\overline{x }\sum_{j=1}^{n}{w}_{ij}}{S\sqrt{\frac{\left[n\sum_{j=1}^{n}{w}_{ij}^{2}-{(\sum_{j=1}^{n}{w}_{ij})}^{2}\right]}{n-1}}}$$

$$\overline{x} = \mathop \sum \limits_{j = 1}^{n} \frac{{x_{j} }}{n};\,S = \sqrt {\frac{{\mathop \sum \nolimits_{j = 1}^{n} x_{j}^{2} }}{n} - \left( {\overline{x}} \right)^{2} }$$

Where ${x}_{j}$ is the attribute value for city *j*, and *n* is the total number of cities; ${w}_{ij}$ is the spatial weight between city *i* and *j*. The first-order queen contiguity spatial weight was used, that is, as long as there was a common edge or the same point between two cities, they were considered to be adjacent, the weight was 1, otherwise the weight was 0. This spatial weight is suitable for modeling some type of infectious disease data [28].

#### Spatial autocorrelation

To explore the spatial association variation over time, global spatial autocorrelation was applied for daily new cases at different time points. *Moran’s I* statistic was chosen as the index of spatial autocorrelation and it has a value range of [− 1,1]. If the value is 0, it means that there is no correlation in different regions, and the incidence is spatially random or independent. Positive *Moran's I* value represents the incidence of disease in adjacent areas is similar, which means a positive correlation. The closer to 1, the stronger the spatial aggregation. Moran's *I* < 0 indicates negative spatial correlation. The smaller the value, the greater the spatial difference among spatial units. Moran’s *I* statistics is given as.

$$Moran^{\prime}s \, I = \frac{{n\sum\nolimits_{j = 1}^{n} {w_{ij} } \left( {x_{j} - \overline{x}} \right){\mkern 1mu} \left( {x_{i} - \overline{x}} \right)}}{{\sum\nolimits_{i \ne j} {w_{ij} } \sum\nolimits_{i} {\left( {x_{i} - \overline{x}} \right)^{2} } }}$$

Where *i*, *j* are city indexes, and ${x}_{j}$ is the number of newly confirmed COVID-19 cases in city j. ${\mathrm{w}}_{\mathrm{ij}}$ is the spatial relationship between city i and j, which was also shown by the first-order queen contiguity spatial weight. *n* represents the number of all space units, namely, the number of cities. $\overline{x }$ is average cases of all cities.

#### Poisson space–time scan statistic

The space–time scanning statistic, proposed by Kulldorff [29]**,** is widely used to search and detect significant clusters of diseases in both space and time, by using dynamically cylindrical moving windows in different time and geographic areas. A space–time cluster can be identified when more cases are observed in the scanning window than expected. The scanning time was set in days and the scanning area was in cities. To avoid extremely large clusters, we tried 10, 20, 25, and 30% of the population at risk respectively as spatial scanning windows. And it was found that the number of cities covered by some clusters exceeded 10–15% of the total number of geographic cities (> 55 cities) when setting 20%-30% of the population at risk as maximum scanning window size [30], which was not appropriated or conducive to disease surveillance. Therefore, in the case of comprehensively weighing the accuracy of the clusters and the actual operability of disease surveillance, the maximum spatial scanning area was set to 10% of the population at-risk, and the maximum scanning time scale was set to 50% of the overall research duration, and the number of Monte Carlo iterations was set to 999. Poisson probability model was used in spatio-temporal scan analysis. According to the principle of Poisson distribution, log-likelihood ratios (*LLR*) of different windows were calculated and were tested by Monte Carlo method to evaluate the statistical significance of space–time cluster. If *P* < 0.05, the relative risk of the cases within the scanning window could be considered to be statistically significant compared with those outside the window. The area with the largest *LLR* value was regarded as the main cluster, and the remaining areas with statistically significant *LLR* values were treated as the secondary clusters. And the spatio-temporal scan results were visualized using ArcGIS 10.3.1.

The log-likelihood ratio of the scan window is.

$$LLR\, = \,\,\frac{{L_{Z} }}{{L_{G} }} = \,\frac{{\left( {\frac{{n_{Z} }}{{\mu_{Z} }}} \right)^{{n_{Z} }} \left( {\frac{{n_{G} - n_{Z} }}{{\mu_{G} - \mu_{Z} }}} \right)^{{n_{G} - n_{Z} }} }}{{\left( {\frac{{n_{G} }}{{\mu_{G} }}} \right)^{{n_{G} }} }}$$

Where ${\mu }_{Z}$ is the expected number of events in the time–space window Z under stochastic assumption; ${\mu }_{G}$ is the total expected number of theoretical events in the whole study time–space range, ${\mu }_{G}=\sum {\mu }_{Z}$, while ${n}_{G}$ is the actual total number of events in the study time–space range.${n}_{Z}$ is the actual number of patients observed in the time–space window Z. Relative risk (RR) was applied to assess the degree of risk of epidemic in each cluster.

$$RR=\frac{{n}_{Z}/{\mu }_{Z}}{\left({n}_{G}-{n}_{Z}\right)/({\mu }_{G}-{\mu }_{Z})}$$

## Results

### Dynamic trend of COVID-19 cases

**Fig. 2:**
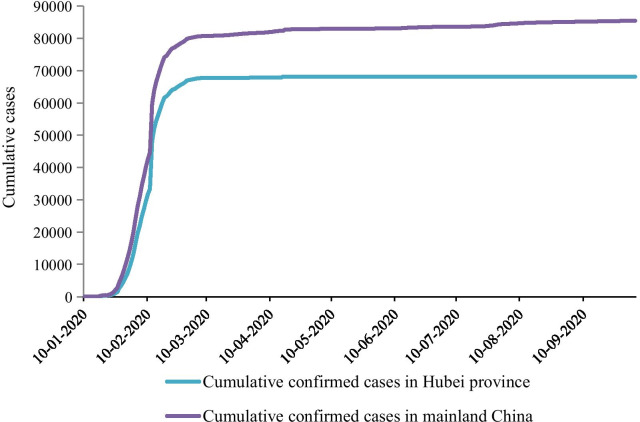
Accumulative confirmed COVID-19 cases in mainland China and Hubei Province from January 10, 2020 to October 5, 2020

**Fig. 3:**
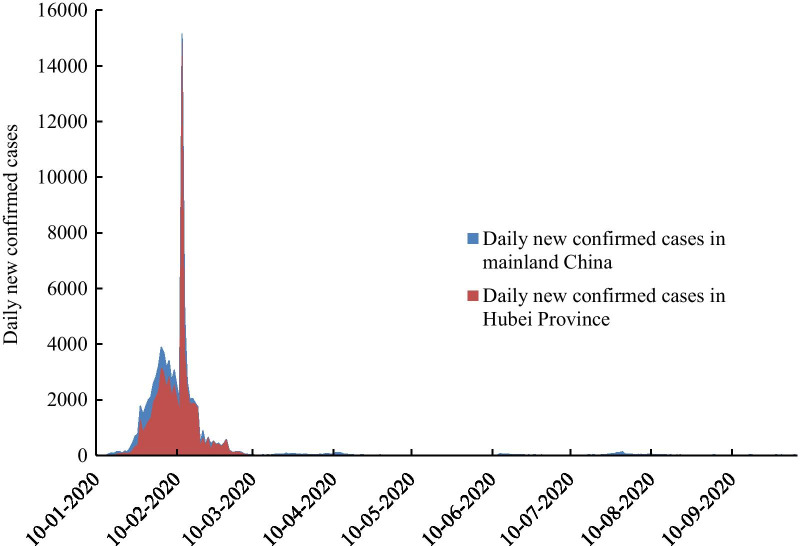
Epidemic curve of COVID-19 cases in mainland China and Hubei Province

As shown in Fig. 2, the overall growth trend of early COVID-19 cases in China was similar to the epidemic situation in Hubei province. After the outbreak of COVID-19 in China at the end of 2019, the number of the cases first started with an explosive rapid increase, then the growth rate began to decrease at the end of February and remained stable after March. Specifically, from January 17 to February 9, 2020, the number of cumulative confirmed cases increased rapidly with the daily increase rate greater than 7.5%. After February 22, the daily increase rate dropped to less than 1%. Since March 6, the number of new cases per day has been less than 100 with sporadic distribution in various regions across the country, except for April 12 and July 28–30. Around mid-March, though the import of overseas cases caused a small increase in the number of cases due to the global epidemic, the COVID-19 cases in China were basically under control as the epidemic in Hubei was curbed. As of October 5, 2020, a total of 85,482 COVID-19 confirmed cases reported in Mainland China, and 79.71% of the COVID-19 cases came from Hubei Province. The epidemic curve based on the diagnosis date (Fig. 3) shows that the number of COVID-19 patients in China and Wuhan Province reached the first epidemic peak from February 3 to February 7, and reached the second peak from February 12 to February 13. As of March 5, the daily new confirmed cases in China were less than 100, and there were no more new cases by mid-March in Hubei.

**Fig. 4:**
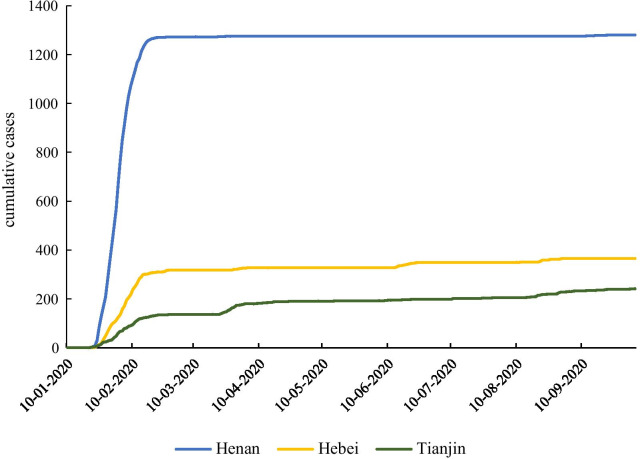
Trend of COVID-19 cases in provinces neighboring Hubei

Figure 4 presents the progress of the COVID-19 epidemic in Henan (adjacent to Hubei province), Hebei (separated from Hubei by one province), and Tianjin (two provinces apart from Hubei). All three regions reported confirmed cases at the end of January, 2020. The increase rate of cases in Henan, a neighboring province of Hubei, was much higher than that in Hebei and Tianjin, suggesting that the spread of the epidemic might be related to the spatial location.

### Spatial epidemic characteristics of COVID-19

**Fig. 5:**
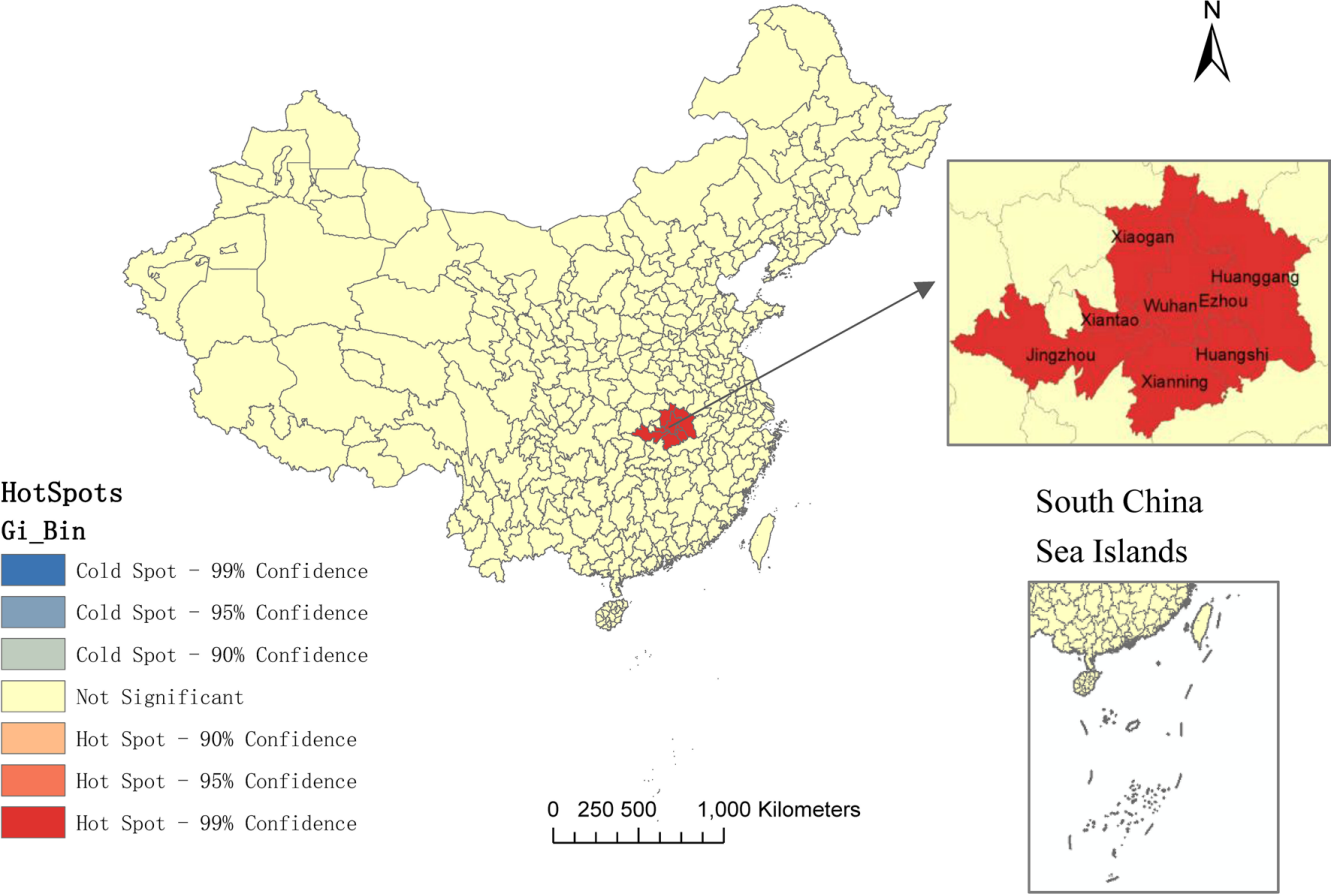
Gi^*^ Cluster Map of COVID-19 cases in mainland China

As of October 5, COVID-19 patients were distributed in 31 provinces and 328 cities (328/362, 90.61%) in Mainland China, of which cases in Hubei Province accounted for 79.71% (68,139/85,482), and 1841 cases in Guangdong Province (2.15%), 1282 cases (1.50%) in Zhejiang Province, and 1281 cases (1.50%) in Henan Province. The cases in Hubei were mainly from Wuhan City (50,344/68,139, 73.88%), while the cases in Guangdong Province mainly distributed in Guangzhou and Shenzhen (1207/1841, 65.56%) cities. Henan patients mainly came from Xinyang, Zhengzhou, Nanyang, and Zhumadian (733/1281, 57.22%). Gi^*^ Cluster Map illustrated that the hot spots for the distribution of COVID-19 cases involved 8 cities in Hubei, including Wuhan, Huangshi, Ezhou, Xiaogan, Jingzhou, Huanggang, Xianning, and Xiantao (Fig. 5).

### Spatiotemporal Pattern of COVID-19

#### Visualization of spatiotemporal changes of COVID-19 cases

**Fig. 6:**
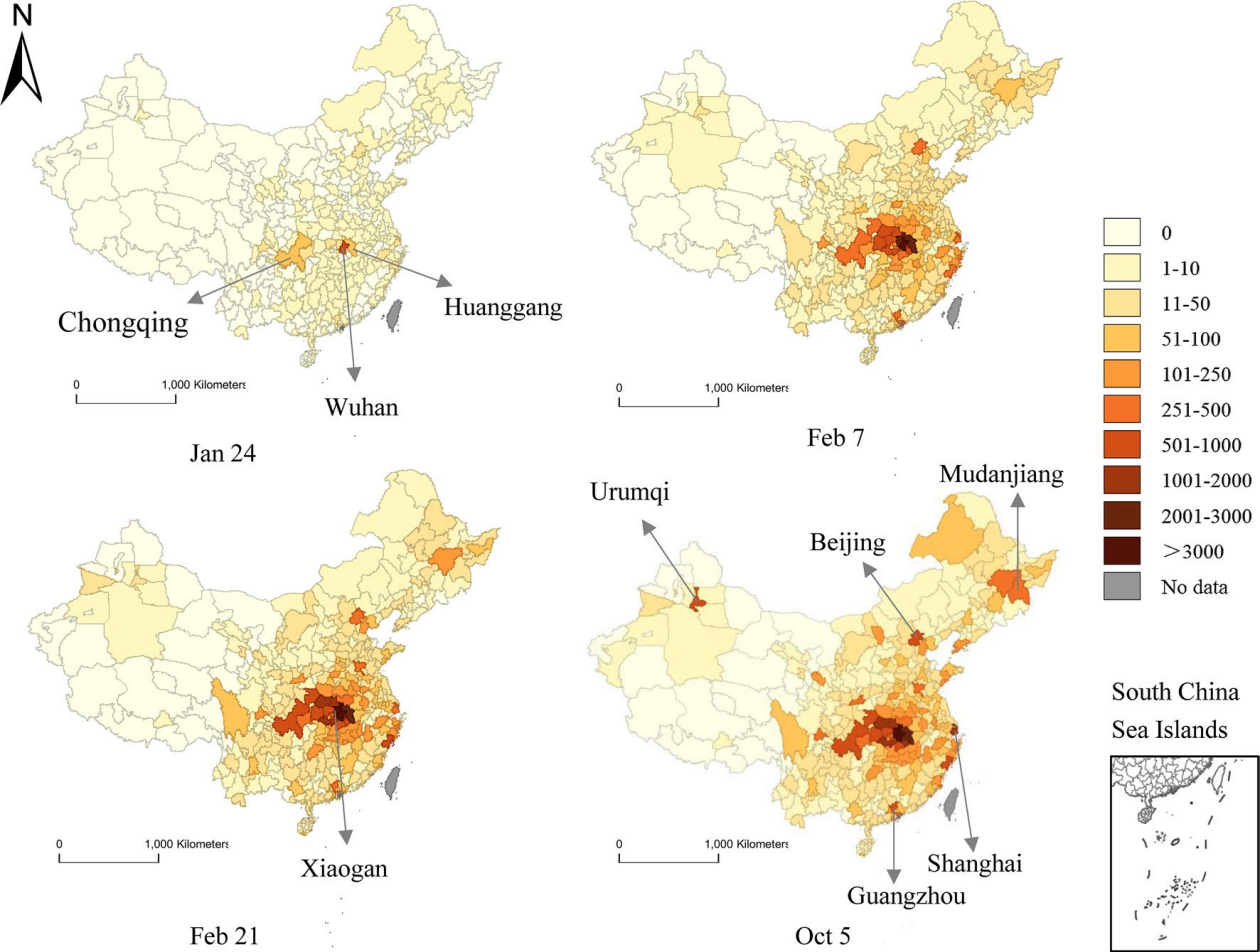
Spatio-temporal distribution of cumulative confirmed COVID-19 cases in mainland China

In order to analyze the temporal and spatial changes of the COVID-19 epidemic in detail, we retrospectively visualized the geographical distribution of the COVID-19 cases in China on January 24, February 7, February 21, and October 5, respectively (Fig. 6). On January 24, 197 (54.42%) cities in China did not report confirmed cases of COVID-19, and 152 (41.99%) cities had 1–10 cases. The city with the highest incidence was Wuhan, with 572 cases, followed by Huanggang City and Chongqing City, with 64 cases and 57 cases respectively. On February 7, the epidemic spread rapidly from Wuhan to other regions, covering 89.50% (324/362) of the cities, especially the cities in Hubei and the surrounding provinces. Over time, on February 21, the number of patients in central and eastern cities in China increased to varying degrees with small changes in western cities, and no confirmed COVID-19 cases were reported in 37 cities. Patients were still mainly distributed in Wuhan, Xiaogan and Huanggang in Hubei Province. As of October 5, the number of patients in most cities remained basically stable. As shown in Fig. 6, only some cities, such as Urumqi, Mudanjiang, Shanghai, Beijing, and Guangzhou, had their maps darkened, that is, the increase in COVID-19 cases was a little obvious in these regions. There were still 34 cities without reported cases. As a supplement, we also paid attention to the incidence rate, and explored the spatio-temporal changes of the incidence of COVID-19 in mainland China, which showed similar spatio-temporal characteristics (Additional file 2).

**Fig. 7:**
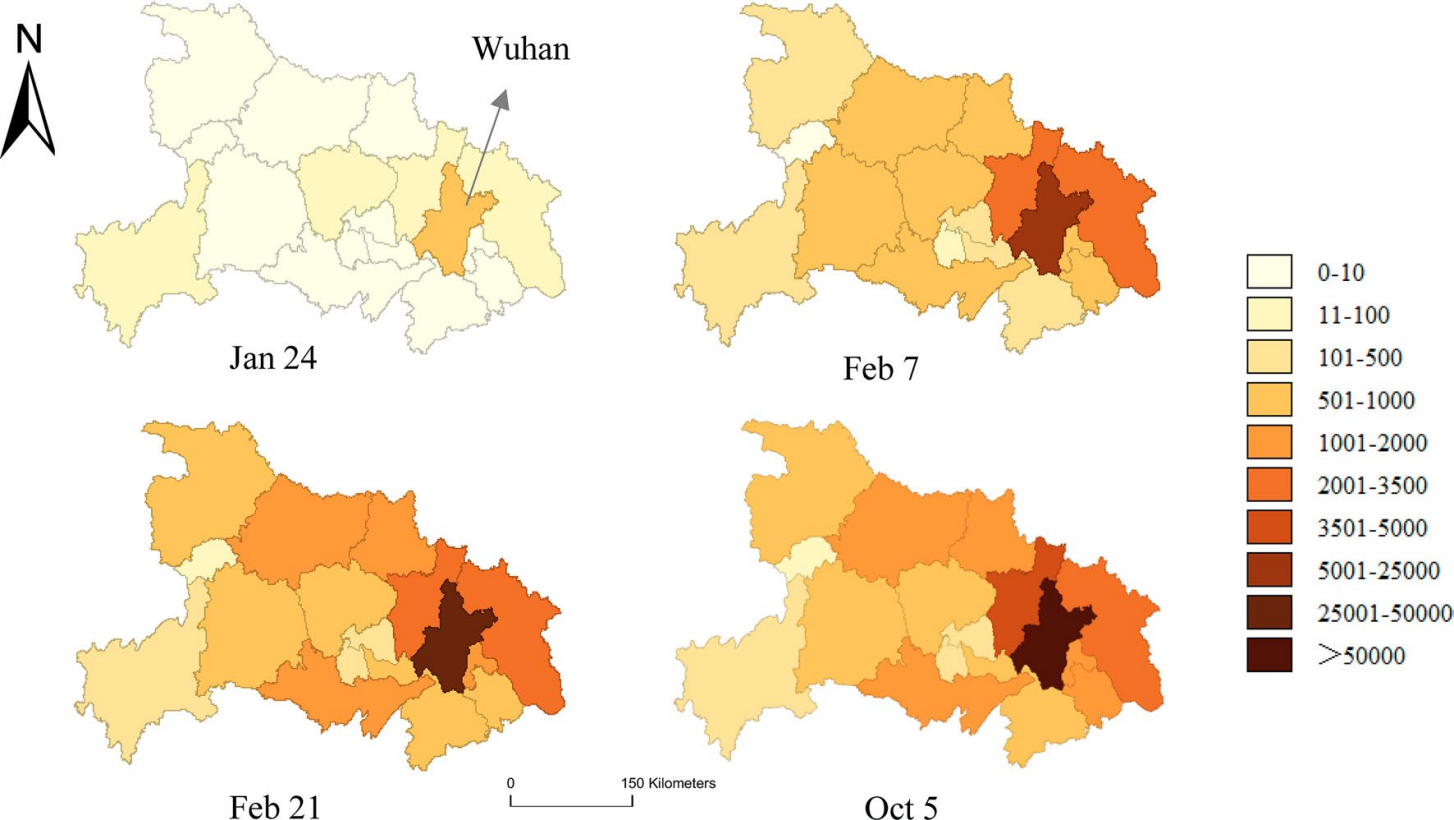
Spatiotemporal distribution of the cumulative confirmed COVID-19 cases in Hubei Province

In Hubei province as displayed in Fig. 7, the COVID-19 cases were only detected in Wuhan at first, and gradually spread to 70.59% (12/17) cities in the province on January 24. On February 7, the epidemic covered the whole province and the number of cases began to increase sharply. As of February 21, the number of cases had increased to about 2.5 times the number of cases on February 7. Then until October 5, the number of cases in other regions did not vary much, except for Wuhan that was still increasing to a certain extent.

#### Dynamics changes of spatial autocorrelation of new COVID-19 cases over time

**Fig. 8:**
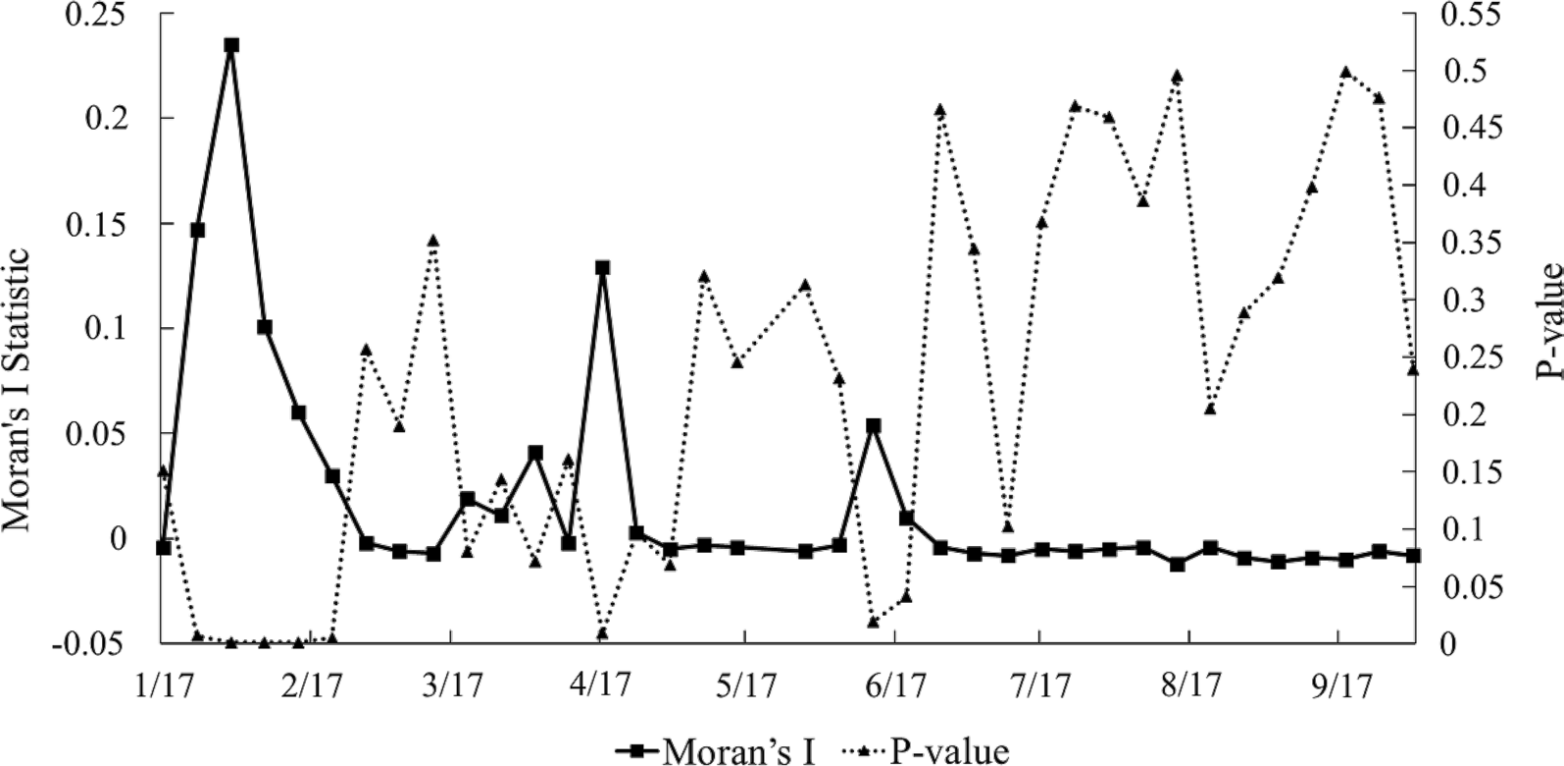
The trends of Moran’s *I* statistic and corresponding *P*-values

The global spatial autocorrelation of daily new COVID-19 cases showed that the spatial correlation increased in the early period and gradually decreased later with a discrete trend to a random distribution. Specifically, as shown in Fig. 8, *Moran’s I* statistic continued to rise after the outbreak, reaching its maximum value on January 31, at 0.235 (*Z* = 12.344, *P* = 0.001), suggesting a moderate spatial aggregation pattern. Then *Moran's I* statistics declined, except for a slight rise around April 17 and June 12 (*P* < 0.05). It suggested that from mid-January to mid-February, mid-April and mid-June, the daily new COVID-19 cases in China were spatially non-random distribution with statistically significant positive correlation to varying degrees. Subsequently, *Moran’s I* statistic dropped and kept at around 0, indicating a decline in clustering, and the COVID-19 cases was gradually distributed randomly in space, but there was no statistical significance (*P* > 0.05).

#### Significant emerging clusters of COVID-19 cases

**Fig. 9:**
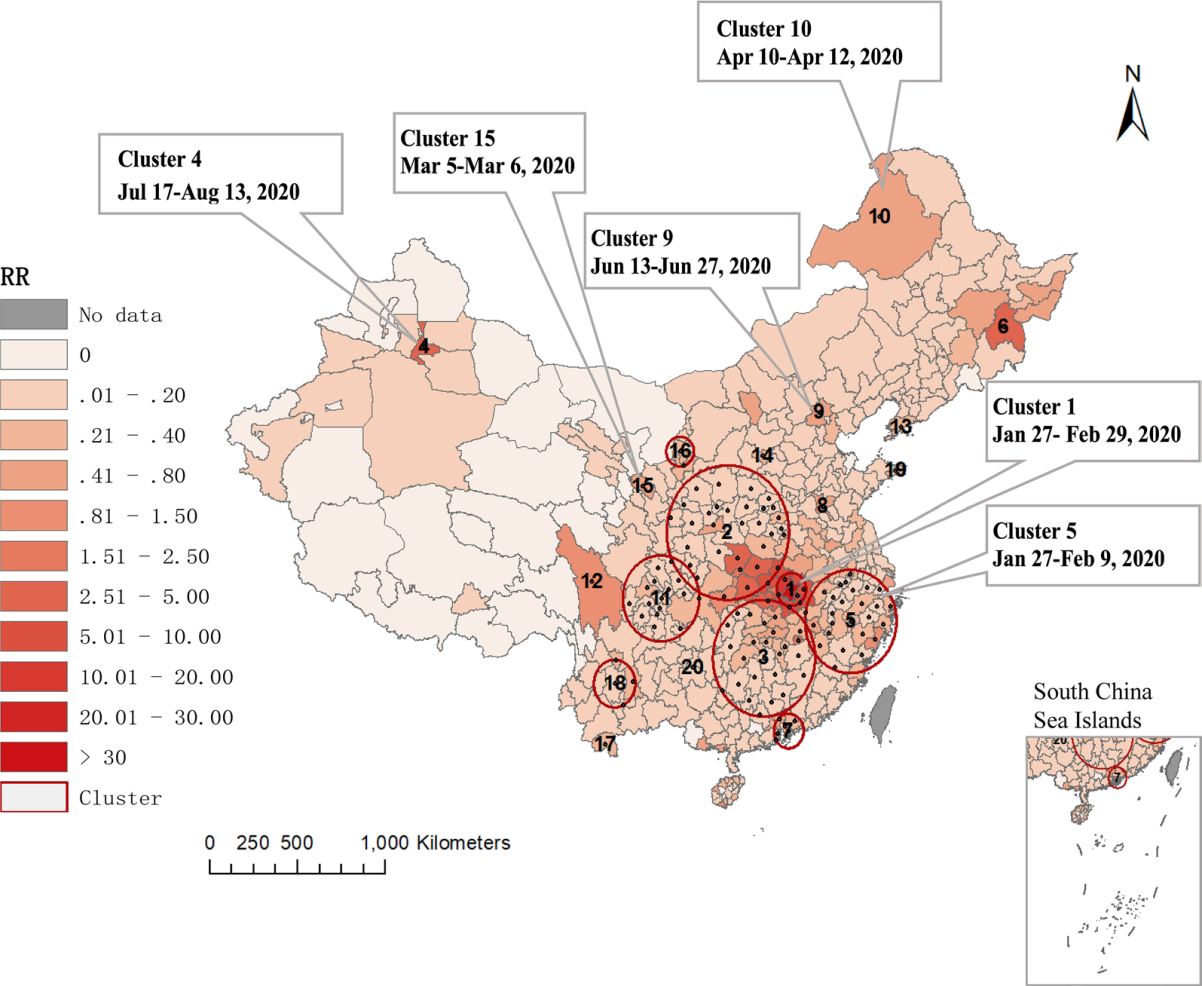
Spatial relative risk distribution of COVID-19 in different cities from January 10 to October 5, 2020

Table 1 summarizes 20 spatiotemporal clusters of COVID-19 cases in mainland China from January 10 to October 5, 2020, at city-level, of which 19 were identified with statistical significance. Most of them (12, 63.16%) occurred from January to February. Clusters lasted from 1 to 34 days. Cluster 1 was the most likely cluster (*RR* = 845.01, *P* < 0.01), including 6 cities in Hubei province with Wuhan as the center. Cluster 2 was located in 33 cities in central China. It had 9.14 times more increased risk of COVID-19 (*RR* = 10.14, *P* < 0.01) compared to other places outside the cluster. Cluster 3 contained 27 cities located south of Wuhan city in south-central China. The population within cluster 3 were at 5.59 times greater risk of COVID-19 (*RR* = 6.59, *P* < 0.01) than regions outside the cluster. Cluster 4 showed that the 811 cases in Urumqi from Jul 17 to Aug 13 were clustered (*RR* = 37.08, *P* < 0.01). More characteristic of each cluster is shown in Table 1. Figure 9 depicts the relative risk of each city and the location of the clusters. Larger clusters were located in central and southern China. Wuhan exhibited the highest *RR* of 178.51, which was also the largest *RR* of the entire analysis process. Out of 362 cities, 34 cities reported a relative risk of 0, while 22 cites had a relative risk greater than 1 (higher observed than expected cases), and 15 cities showed a relative risk greater than 3.

**Table 1:** Emerging space–time cluster of COVID-19 in mainland China from January 10 to October 5, 2020, at city-level

Cluster	Duration(days)	Center	Radius	# of cities	Observed	Expected	*RR*	*LLR*	*P*
1	Jan 27–Feb 29	Wuhan	102.46	6	57,505	207.52	845.01	292,260.15	< 0.01
2	Jan 25–Feb 13	Shangluo	408.41	33	5674	595.26	10.14	7869.28	< 0.01
3	Jan 25–Feb 14	Hengyang	366.96	27	3663	576.47	6.59	3743.56	< 0.01
4	Jul 17–Aug 13	Urumqi	0.00	1	811	22.08	37.08	2137.36	< 0.01
5	Jan 27–Feb 9	Quzhou	322.25	26	1632	339.54	4.88	1279.59	< 0.01
6	Apr 4–Apr 17	Mudanjiang	0.00	1	368	7.92	46.68	1053.46	< 0.01
7	Jan 26–Feb 8	Shenzhen	110.91	6	844	149.54	5.69	769.00	< 0.01
8	Feb 20	Jining	0.00	1	201	1.88	107.21	740.27	< 0.01
9	Jun 13–Jun 27	Beijing	0.00	1	304	72.65	4.20	204.09	< 0.01
10	Apr 10–Apr 12	Hulunbeir	0.00	1	62	1.71	36.29	162.36	< 0.01
11	Jan 24–Feb 8	Ziyang	266.53	16	705	351.38	2.01	138.03	< 0.01
12	Feb 3–Feb 27	Tibetan Autonomous Prefecture of Garze	0.00	1	72	6.74	10.69	105.29	< 0.01
13	Jul 24–Aug 2	Dalian	0.00	1	84	15.73	5.34	72.48	< 0.01
14	Apr 7	Taiyuan	0.00	1	25	1.00	24.92	56.39	< 0.01
15	Mar 5–Mar 6	Lanzhou	0.00	1	28	1.71	16.43	52.07	< 0.01
16	Jan 26–Feb 14	Yinchuan	87.90	2	59	16.52	3.57	32.63	< 0.01
17	Jan 28–Jan 30	Sipsongpanna	0.00	1	11	0.80	13.72	18.61	< 0.01
18	Jan 28–Feb 1	Chuxiong Yi Autonomous Prefecture	150.77	4	43	14.97	2.87	17.36	0.02
19	Jan 26–Jan 31	Weihai	0.00	1	20	3.83	5.23	16.90	0.03
20	Feb 11	Qiannan Buyi and Miao Autonomous Prefecture	0.00	1	9	0.74	12.16	14.22	0.29

*RR* Relative Risk, *LLR* Log-likelihood ratio

## Discussion

This city-level modeling study analyzed and visualized the spatio-temporal distribution characteristics of COVID-19 in mainland China from perspectives of time, space, and space–time, respectively, to explore the high-risk hot spots and systematically discuss the space–time aggregation pattern of COVID-19 at the city level across the country, which could provide scientific reference for the allocation of medical resources in areas with different epidemic levels. Also, this study could support the healthcare facilities in monitoring and early warning of COVID-19 or other epidemics of the same type, as well as alerting to the potential rebound of the epidemic in a certain area.

In terms of time alone, the high incidence duration of China’s COVID-19 epidemic was from January 17 to February 9, 2020, as the daily increase rate was greater than 7.5%, which was a signal of the epidemic and outbreak. The first wave of COVID-19 outbreak in China lasted for about 20 days [31]. A study pointed out that the number of daily new cases reached a peak between February 3 and 9 with a spike on February 12, 2020, showing time clustering, which was consistent to our result [32]. In response to the epidemic, almost all countries implemented different lockdown policies to alleviate the spreading of the disease [16, 33]. Due to the large population and active socio-economic vitality, China was facing greater pressure from the COVID-19 outbreak. Therefore, on January 26, 2020, 30 provinces in China initiated the first-level response to public health emergencies [34]. China launched an unprecedented strict lockdown policy including the prohibition of unnecessary commercial activities in daily lives, urging people to stay at home to prevent any kind of people gathering, and establishing restrictions on public transportation [31]. Around mid-March, the COVID-19 epidemic in China was basically under control. These rigorous prevention and control measures were proven to take into effect in restricting COVID-19 spreading speed and mitigating the burden of both medical resources and costs [21, 31, 35].

From a spatial perspective, the hot spot analysis based on the number of cases suggested that the cities including Wuhan, Huangshi, Ezhou, Xiaogan, Jingzhou, Huanggang, Xianning, and Xiantao, were the hot spots with statistical significance in the distribution of COVID-19 cases in China, which was similar to the results of cluster 1 in the spatio-temporal scan statistic. Gi^*^ Cluster Map based on incidence rate showed the similar hot spots, which can be a supplementary verification to the existing results (Additional file 2). Almost all hotspots were cities adjacent to Wuhan, which might be attributed to their frequent cross-regional social and economic activities and connections with Wuhan. After the outbreak, more than 5 million people emigrated from Wuhan before the city blockade [10]. A study suggested that most of the early cases had a history of travel or residence in Wuhan, then became the main source of infection in other cities [36]. Studies showed that the progress of the COVID-19 epidemic was related to population migration [9], especially the population movement from Hubei, and the reported cases in various regions were positively correlated with the population migration index [37]. With the identification of hot spots, prevention and control measures such as publicity, education, monitoring, and training should be carried out in high-risk areas [38]. Spatial autocorrelation analysis indicated a moderately correlated pattern of spatial clustering of COVID-19 cases across China in the early stage. However, the spatial correlation gradually decreased in the later period and showed a discrete and random trend. This result was similar to one study, which revealed that the COVID-19 cases were positively correlated in space before February 3 in China, and gradually decreased, then distributed randomly after February 11 [39]. To a certain extent, the reduction of spatial correlation also implied that the series of critical actions taken by China for suppressing the outbreak were effective [32, 40].

From the spatiotemporal perspective, based on space–time scan statistic, we found most clusters happened in January and February and were located in central and southern China. Cluster 1 was discovered at the end of January, which was also the one where the epidemic lasted the longest. With the discovery of cluster 1, China began to implement school closures, travel restrictions, community-level lockdown, and contact tracing around late January [41]. Cluster 2, 3, 5, 7, 11 and 16 were also occurred in the early phase and ended in mid-February. Most of them were located around cluster 1, and the communication activities with cluster 1 were highly active. The cluster 15 that occurred in Lanzhou in early March and the Cluster 6, 10 and 14 that occurred around mid-April were cities with large international airports, which were caused by a surge in imported cases from abroad. Therefore, starting from March, China shifted the focus of measures to the testing and quarantine of inbound passengers to maintain control of the disease [39]. Cluster 9 broke out locally in Beijing in June. As the capital, Beijing has developed to one of the centres of transportation and economy in China and even around the world. Many studies showed that frequent economic and trade activities could supply a way for new viruses to spread over long distances, which might increase the risk of infectious disease outbreaks [42, 43]. Cluster 13 was formed by the continuous importation of overseas cases from the end of July to the beginning of August. Cluster 4 was the aggregation of Xinjiang local cases from July 17 to August 13. There had no emerging spatiotemporal clusters were detected in September. Overall, the clusters with larger coverage were in the early phase of the epidemic, while it changed to only gather in a specific city in the later period. The scope of the cluster was shrinking, implying that China’s strict prevention and control measures on population movement played a role and had practical implications.

The spatio-temporal distribution characteristics of diseases are related to the course of the epidemic, which presents obvious clustering during the epidemic period [12]. Therefore, the clustering detection in time and space is crucially important in the exploration and early warning of disease outbreak and recurrence. To our knowledge, this research was the first to analyze the spatial and temporal distribution characteristics of COVID-19 on the city level from a national perspective in mainland China, which could play a pivotal role in assessing the level of epidemics in different cities, optimizing the allocation of medical resources, evaluating the effects of prevention and control interventions, and assisting in making healthcare decisions. For instance, this study could effectively provide public health officials and decision-makers with information on the spatiotemporal spread of COVID -19 disease in time, and keep them well-informed on when and where to improve the allocation of resources and testing sites, and to implement stricter quarantine and travel bans. Based on this study, further monitoring the spatial clustering pattern of COVID-19 and detecting the emergence of new clusters or the enhancement of existing clusters can, from city level, recognize potential epidemic rebounds and achieve early prevention and control of clusters.

Although our findings give a helpful insight into the spatio-temporal distribution characteristics of COVID-19 in China, this study has some limitations. Firstly, our research was based on confirmed cases, and the suspected cases and cases diagnosed as positive but asymptomatic were not included according to the definition, so the true magnitude of the COVID-19 epidemic needed to be interpreted dialectically. Secondly, the latest number of permanent residents in 2019 was used in the space–time scan analysis, which might differ with the local population in 2020, so the research results might be biased. Thirdly, this study utilized COVID-19 case data as of October 5, 2020, and did not further collect COVID-19 cases in winter. Studies showed that the spreading and transmission rate of COVID-19 could significantly positively associate with temperature [21, 31, 44], so there might be emerging clusters that reappear in winter. However, a study held the opposite view that mean temperature and relative humidity were inversely associated with COVID-19 growth curve in Africa [45]. Another study suggested there was no evidence that COVID-19 cases decreased in warm temperatures [46]. Therefore, it is still necessary to further investigate whether there are new clusters in China during the winter. Besides, in space–time scan statistics, confidence interval for the relative risk of detected clusters was not included, since the estimation method is still an open challenge, but is an interesting research worthy of in-depth exploration in future. Additionally, for the current study, due to the lack of a unified open sharing platform to realize data acquisition and sharing at city level, we spent a lot of energy and time in the data collation. Thus, barriers to data openness and sharing will be a bottleneck that needs to be resolved for monitoring an epidemic and exploring further research and formulating policies to respond to infectious disease in the future [41].

## Conclusion

This study provides an overview of the spatiotemporal clustering pattern and hot spots of COVID-19 cases in China at the city level. Overall, the clusters with larger coverage emerged in the early phase of the epidemic, while they changed to only gather in a specific city in the later period, which showed the pattern and scope of clusters changed and reduced over time. The strict prevention and control strategies adopted by Chinese government were effective to cope with the COVID-19 epidemic. However, persistent efforts need to be made to prevent the rebound of the epidemic, especially in the context of the global pandemic. Analyzing spatial and temporal distribution characteristics of COVID-19 cases for different cities across the country is useful to assess the level of epidemics and evaluate the effects of prevention and control measures in different regions. The research method can be further used to monitor the changes in spatial clustering pattern of COVID-19 cases and detect the emergence of new clusters, and then provide useful clues for healthcare facilities to quickly respond to the highly changeable and contagious epidemics.

## Supplementary Information

**Additional file 1:** The list of research cities.

**Additional file 2:Fig. S1.** Gi* Cluster Map of COVID-19 incidence in mainland China. **Fig. S2.** Spatio-temporal distribution of the incidence of COVID-19 in mainland China.

## Data Availability

The datasets were collected from daily notification of the National Health Commission of China(http://www.nhc.gov.cn/xcs/yqtb/list_gzbd.shtml) and provincial Health Commissions (eg. http://wst.hainan.gov.cn/swjw/rdzt/yqfk/index_3.html). The datasets used and analyzed during the study are available from the corresponding author on reasonable request.

## References

[1] Li Q, Guan X, Wu P (2020). Early transmission dynamics in Wuhan, China, of novel coronavirus-infected pneumonia. New Engl J Med.

[2] Ayittey FK, Dzuvor C, Ayittey MK (2020). Updates on Wuhan 2019 novel coronavirus epidemic. J Med Virol.

[3] Chang D, Lin M, Wei L (2020). Epidemiologic and clinical characteristics of novel coronavirus infections involving 13 patients outside Wuhan, China. JAMA.

[4] Gralinski LE, Menachery VD (2020). Return of the coronavirus: 2019-nCoV. Viruses.

[5] Tang B, Bragazzi NL, Li Q (2020). An updated estimation of the risk of transmission of the novel coronavirus (2019-nCov). Infect Dis Model.

[6] Lu R, Zhao X, Li J (2020). Genomic characterisation and epidemiology of 2019 novel coronavirus: implications for virus origins and receptor binding. Lancet.

[7] Lai C, Shih T, Ko W (2020). Severe acute respiratory syndrome coronavirus 2 (SARS-CoV-2) and coronavirus disease-2019 (COVID-19): the epidemic and the challenges. Int J Antimicrob Ag.

[8] Ortiz-Prado E, Simbaña-Rivera K, Gómez-Barreno L (2020). Clinical, molecular, and epidemiological characterization of the SARS-CoV-2 virus and the coronavirus disease 2019 (COVID-19), a comprehensive literature review. Diagn Microbiol Infect Dis.

[9] Chan JF, Yuan S, Kok K (2020). A familial cluster of pneumonia associated with the 2019 novel coronavirus indicating person-to-person transmission: a study of a family cluster. Lancet.

[10] Mo C, Tan D, Mai T (2020). An analysis of spatiotemporal pattern for COIVD-19 in China based on space-time cube. J Med Virol.

[11] Cohen J (2020). New coronavirus threat galvanizes scientists. Science.

[12] Desjardins MR, Hohl A, Delmelle EM (2020). Rapid surveillance of COVID-19 in the United States using a prospective space-time scan statistic: Detecting and evaluating emerging clusters. Appl Geogr.

[13] WHO. The World Health Organization Coronavirus Disease 2019 (COVID-19) Situation Report. 2020; https://www.who.int/emergencies/diseases/novel-coronavirus-2019. Accessed 23 Oct 2020.

[14] Chinese Center for Disease Control and Prevention. The situation and distribution of the COVID-19 epidemic from the Chinese Center for Disease Control and Prevention [In Chinese]. 2020; http://2019ncov.chinacdc.cn/2019-nCoV/. Accessed 23 Oct 2020.

[15] Fu L, Wang B, Yuan T (2020). Clinical characteristics of coronavirus disease 2019 (COVID-19) in China: a systematic review and meta-analysis. J Infect.

[16] Yeşilkanat CM (2020). Spatio-temporal estimation of the daily cases of COVID-19 in worldwide using random forest machine learning algorithm. Chaos Solitons Fractals.

[17] Fu L, Wang B, Yuan T (2020). Clinical characteristics of coronavirus disease 2019 (COVID-19) in China: a systematic review and meta-analysis. J Infection.

[18] Hu J, Zhang Y, Wang W (2021). Clinical characteristics of 14 COVID-19 deaths in Tianmen, China: a single-center retrospective study. BMC Infect Dis.

[19] Kang D, Choi H, Kim JH (2020). Spatial epidemic dynamics of the COVID-19 outbreak in China. Int J Infect Dis.

[20] Zhang X, Rao H, Wu Y (2020). Comparison of spatiotemporal characteristics of the COVID-19 and SARS outbreaks in Mainland China. BMC Infect Dis.

[21] He J, Chen G, Jiang Y (2020). Comparative infection modeling and control of COVID-19 transmission patterns in China, South Korea Italy and Iran. Sci Total Environ.

[22] Adekunle IA, Onanuga AT, Akinola OO (2020). Modelling spatial variations of coronavirus disease (COVID-19) in Africa. Sci Total Environ.

[23] National Health Commission of the People’s Republic of China. Novel coronavirus pneumonia epidemic prevention and control-epidemic notification, 2020; http://www.nhc.gov.cn/xcs/yqtb/list_gzbd.shtml. Accessed 20 Oct 2020.

[24] General Office of the National Health Commission of China, Notice on issuing the COVID-19 diagnosis and treatment plan. 2020; http://www.nhc.gov.cn/yzygj/s7653p/202008/0a7bdf12bd4b46e5bd28ca7f9a7f5e5a.shtml. Accessed 20 Apr 2021.

[25] National Geomatics Center of China. National basic geographic information database. 2020; http://www.ngcc.cn/ngcc/. Accessed 20 Oct 2020.

[26] ESRI. How Hot Spot Analysis (Getis-Ord Gi*) works. 2020; https://desktop.arcgis.com/en/arcmap/10.3/tools/spatial-statistics-toolbox/h-how-hot-spot-analysis-getis-ord-gi-spatial-stati.htm? Accessed 20 Oct 2020.

[27] Ord JK, Getis A (1995). Local spatial autocorrelation statistics: distributional issues and an application. Geogr Anal.

[28] ESRI. ArcGIS desktop help. 2009; http://webhelp.esri.com/arcgisdesktop/9.3/index.cfm?TopicName=Modeling_spatial_relationships. Accessed 20 Oct 2020.

[29] Kulldorff M, Huang L, Pickle L (2010). An elliptic spatial scan statistic. Stat Med.

[30] Tango T, Takahashi K (2005). A flexibly shaped spatial scan statistic for detecting clusters. Int J Health Geogr.

[31] Sun Z, Zhang H, Yang Y (2020). Impacts of geographic factors and population density on the COVID-19 spreading under the lockdown policies of China. Sci Total Environ.

[32] Xiao J, Hu J, He G (2020). The time-varying transmission dynamics of COVID-19 and synchronous actions in China. Int J Infect Dis.

[33] Jeong GH, Lee HJ, Lee J (2020). Effective control of COVID-19 in South Korea: cross-sectional study of epidemiological data. J Med Internet Res.

[34] The State Council Information Office of China. The State Council Information Office held a press conference on joint prevention and control of COVID-19 disease. 2020. http://www.scio.gov.cn/xwfbh/xwbfbh/wqfbh/42311/42478/index.htm. Accessed 20 Oct 2020.

[35] Pan A, Liu L, Wang C (2020). Association of public health interventions with the epidemiology of the COVID-19 outbreak in Wuhan. China JAMA.

[36] Chen ZL, Zhang Q, Lu Y (2020). Distribution of the COVID-19 epidemic and correlation with population emigration from Wuhan, China. Chin Med J (Engl).

[37] Hu J, He G, Tao L (2020). Risk assessment of exported risk of COVID-19 from Hubei Province (in Chinese). Chin J Prev Med.

[38] Cui T, Yang G, Ji L (2020). Chinese residents' perceptions of COVID-19 during the pandemic: online cross-sectional survey study. J Med Internet Res.

[39] Su L, Wen G (2020). Spatial aggregation and spatial-temporal pattern of provincial cumulative confirmed count of novel coronavirus pneumonia (COVID-19) in China (in Chinese). J Chongqing Inst Tech.

[40] Li Z, Chen Q, Feng L (2020). Active case finding with case management: the key to tackling the COVID-19 pandemic. Lancet.

[41] Fu H, Wang H, Xi X (2020). A database for the epidemic trends and control measures during the first wave of COVID-19 in mainland China. Int J Infect Dis.

[42] Ceddia MG, Bardsley NO, Goodwin R (2013). A complex system perspective on the emergence and spread of infectious diseases: Integrating economic and ecological aspects. Ecol Econ.

[43] Findlater A, Bogoch II (2018). Human mobility and the global spread of infectious diseases: a focus on air travel. Trends ParasitoL.

[44] Guo C, Bo Y, Lin C (2020). Meteorological factors and COVID-19 incidence in 190 countries: an observational study. Sci Total Environ.

[45] Adekunle IA, Tella SA, Oyesiku KO (2020). Spatio-temporal analysis of meteorological factors in abating the spread of COVID-19 in Africa. Heliyon..

[46] Briz-Redón Á, Serrano-Aroca Á (2020). A spatio-temporal analysis for exploring the effect of temperature on COVID-19 early evolution in Spain. Sci Total Environ.

