# Supplementary material for: Spatio-temporal distribution characteristics of COVID-19 in China: a city-level modeling study
Source: BMC Infect Dis. 2021 Aug 14;21:816. doi: 10.1186/s12879-021-06515-8 (PMC8363872; doi:10.1186/s12879-021-06515-8)
Supplement: Supplementary file 1 — Additional file 1: The list of research cities. [file 12879_2021_6515_MOESM1_ESM.pdf]

Additional file 1: The list of research cities

Province/municipalities/autonomous regions	# of cities	Name of the cities
Beijing	1	Beijing
Tianjin	1	Tianjin
Hebei	11	Shijiazhuang, Tangshan, Qinhuangdao, Handan, Xingtai, Baoding, Zhangjiakou, Chengde, Cangzhou, Langfang, Hengshui
Shānxi	11	Taiyuan, Datong, Yangquan, Changzhi, Jincheng, Shuozhou, Jinzhong, Yuncheng, Xinzhou, Linfen, Lvliang
Inner Mongolia	12	Hohhot, Baotou, Wuhai, Chifeng, Tongliao, Ordos, Hulun Buir, Bayan Nur, Ulanqab, Hinggan League, Xilingol League, Alxa League
Liaoning	14	Shenyang, Dalian, Anshan, Fushun, Benxi, Dandong, Jinzhou, Yingkou, Fuxin, Liaoyang, Panjin, Tieling, Chaoyang, Huludao
Jilin	11	Changchun, Jilin, Siping, Gongzhuling, Liaoyuan, Tonghua, Meihekou, Baishan, Songyuan, Baicheng, Yanbian Korean Autonomous Prefecture
Heilongjiang	13	Harbin, Qiqihar, Jixi, Hegang, Shuangyashan, Daqing, Yichun, Jiamusi, Qitaihe, Mudanjiang, Heihe, Suihua, Daxinganling,
Shanghai	1	Shanghai
Jiangsu	13	Nanjing, Wuxi, Xuzhou, Changzhou, Suzhou, Nantong, Lianyungang, Huaian, Yancheng, Yangzhou, Zhenjiang, Taizhou, Suqian,
Zhejiang	11	Hangzhou, Ningbo, Wenzhou, Jiaxing, Huzhou, Shaoxing, Jinhua, Quzhou, Zhoushan, Taizhou, Lishui,
Anhui	16	Hefei, Wuhu, Bengbu, Huainan, Ma'anshan, Huaibei, Tongling, Anqing, Huangshan, Chuzhou, Fuyang, Suzhou, Lu'an, Haozhou, Chizhou, Xuancheng

Province/municipalities/autonomous regions	# of cities	Name of the cities
Fujian	9	Fuzhou, Xiamen, Putian, Sanming, Quanzhou, Zhangzhou, Nanping, Longyan, Ningde,
Jiangxi	11	Nanchang, Jingdezhen, Pingxiang, Jiujiang, Xinyu, Yingtan, Ganzhou, Ji'an, Yichun, Fuzhou, Shangrao
Shandong	16	Jinan, Qingdao, Zibo, Zaozhuang, Dongying, Yantai, Weifang, Jining, Tai'an, Weihai, Rizhao, Linyi, Dezhou, Liaocheng, Binzhou, Heze
Henan	18	Zhengzhou, Kaifeng, Luoyang, Pingdingshan, Anyang, Hebi, Xinxiang, Jiaozuo, Puyang, Xuchang, Luohe, Sanmenxia, Nanyang, Shangqiu, Xinyang, Zhoukou, Zhumadian, Jiyuan
Hubei	17	Wuhan, Huangshi, Shiyan, Yichang, Xiangfan, Ezhou, Jingmen, Xiaogan, Jingzhou, Huanggang, Xianning, Suizhou, Enshi Tujia and Miao Autonomous Prefecture, Xiantao, Qianjiang, Tianmen, Shennongjia Forestry District
Hunan	14	Changsha, Zhuzhou, Xiangtan, Hengyang, Shaoyang, Yueyang, Changde, Zhangjiajie, Yiyang, Binzhou, Yongzhou, Huaihua, Loudi, Xiangxi Tujia and Miao Autonomous Prefecture
Guangdong	21	Guangzhou, Shaoguan, Shenzhen, Zhuhai, Shantou, Foshan, Jiangmen, Zhanjiang, Maoming, Zhaoqing, Huizhou, Meizhou, Shanwei, Heyuan, Yangjiang, Qingyuan, Dongguan, Zhongshan, Chaozhou, Jieyang, Yunfu
Guangxi	14	Nanning, Liuzhou, Guilin, Wuzhou, Beihai, Fangchenggang, Qinzhou, Guigang, Yulin, Baise, Hezhou, Hechi, Laibin, Chongzuo

Province/municipalities/autonomous regions	# of cities	Name of the cities
Haikou	18	Haikou, Sanya, Danzhou, Wuzhishan, Qionghai, Wengchang, Wanning, Dongfang, Dingan, Tunchang, Chengmai, Lingao, Baisha, Changjiang, Ledong, Lingshui, Baoting, Qiongzhong
Chongqing	1	Chongqing
Sichuan	21	Chengdu, Zigong, Panzhihua, Luzhou, Deyang, Mianyang, Guangyuan, Suining, Neijiang, Leshan, Nanchong, Meishan, Yibin, Guangan, Dazhou, Yaan, Bazhong, Ziyang, Tibetan Qiang Autonomous Prefecture of Ngawa, Tibetan Autonomous Prefecture of Garzê, Liangshan Yi Autonomous Prefecture
Guizhou	9	Guiyang, Lupanshui, Zunyi, Anshun, Tongren, Qianxi'nan Buyi and Miao Autonomous Prefecture, Bijie, Qiandongnan Miao and Dong Autonomous Prefecture, Qiannan Buyi and Miao Autonomous Prefecture
Yunnan	16	Kunming, Qujing, Yuxi, Baoshan, Zhaotong, Lijiang, Puer, Lincang, Chuxiong, Hani-Yi Autonomous Prefecture of Honghe, Wenshan Zhuang and Miao Autonomous Prefecture, Sipsongpanna, Dali Bai Autonomous Prefecture, Dehong Dai and Jingpo Autonomous Prefecture, Nujiang of the Lisu Autonomous Prefecture, Diqing Tibetan Autonomous Prefecture
Tibet	7	Lhasa, Qamdo, Shannan, Xigaze, Nagqu, Ngari, Nyingchi
Shǎnxi	10	Xi'an, Tongchuan, Baoji, Xianyang, Weinan, Yanan, Hanzhong, Yulin, Ankang, Shangluo

**Province/municipalities/autonomous regions**	**# of cities**	**Name of the cities**
Gansu	14	Lanzhou, Jiayuguan, Jinchang, Baiyin, Tianshui, Wuwei, Zhangye, Pingliang, Jiuquan, Qingyang, Dingxi, Longnan, Linxia Hui Autonomous Prefecture, Gannan Tibetan Autonomous Prefecture
Qinghai	8	Xining, Haidong, Tibetan Autonomous Prefecture of Haibei, Tibetan Autonomous Prefecture of Huangnan, Tibetan Autonomous Prefecture of Hainan, Tibetan Autonomous Prefecture of Golog, Yushu Tibetan Autonomous Prefecture, Haixi Mongolian and Tibetan Autonomous Prefecture
Ningxia	5	Yinchuan, Shizuishan, Wuzhong, Guyuan, Zhongwei
Xinjiang	18	Urumqi, Karamay, Turpan, Hami, Changji Hui Autonomous Prefecture, Bortala Mongol Autonomous Prefecture, Bayingol Mongolian Autonomous Prefecture, Aksu, Kizilsu Kirghiz Autonomous Prefecture, Kashgar, Hotan, Ili Kazak Autonomous Prefecture, Tacheng, Altay, Shihezi, Alaer, Tumushuke, Wujiaqu
