# Supplementary material for: Spatio-temporal distribution characteristics of COVID-19 in China: a city-level modeling study
Source: BMC Infect Dis. 2021 Aug 14;21:816. doi: 10.1186/s12879-021-06515-8 (PMC8363872; doi:10.1186/s12879-021-06515-8)
Supplement: Supplementary file 2 — Additional file 2:Fig. S1. Gi* Cluster Map of COVID-19 incidence in mainland China. Fig. S2. Spatio-temporal distribution of the incidence of COVID-19 in mainland China. [file 12879_2021_6515_MOESM2_ESM.pdf]

Additional file 2

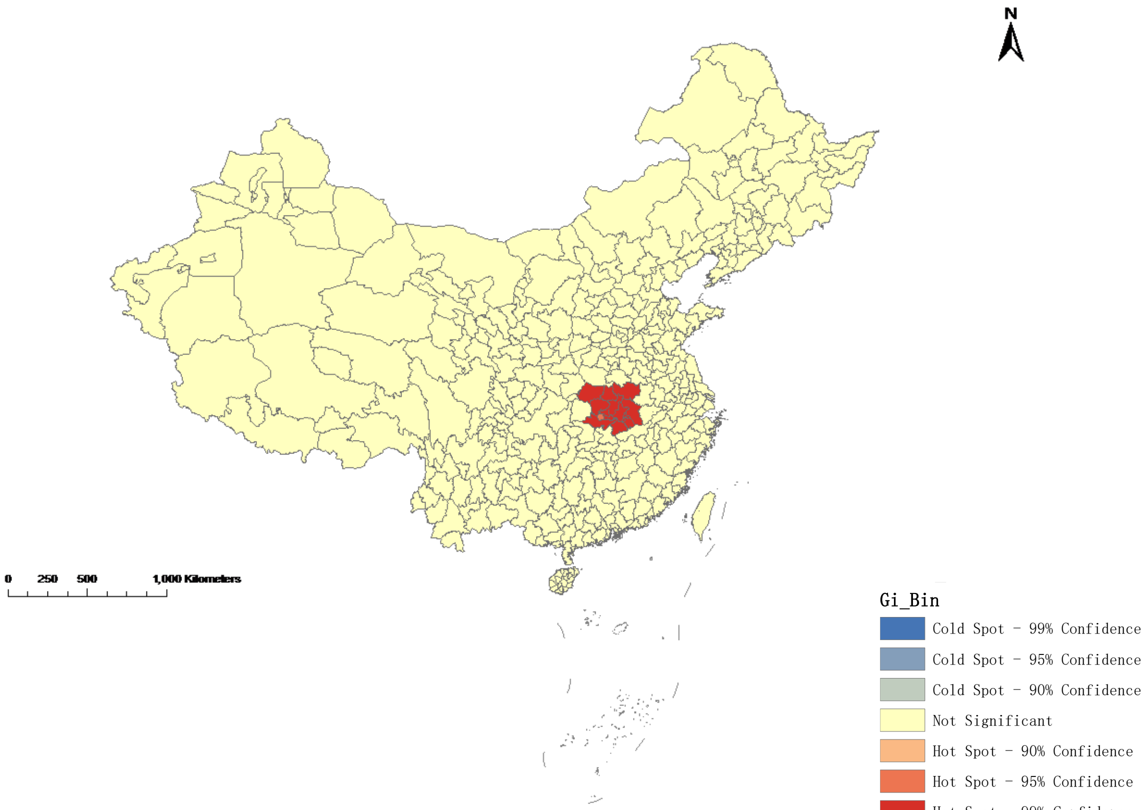

Figure 1 Gi* Cluster Map of COVID-19 incidence in Mainland China

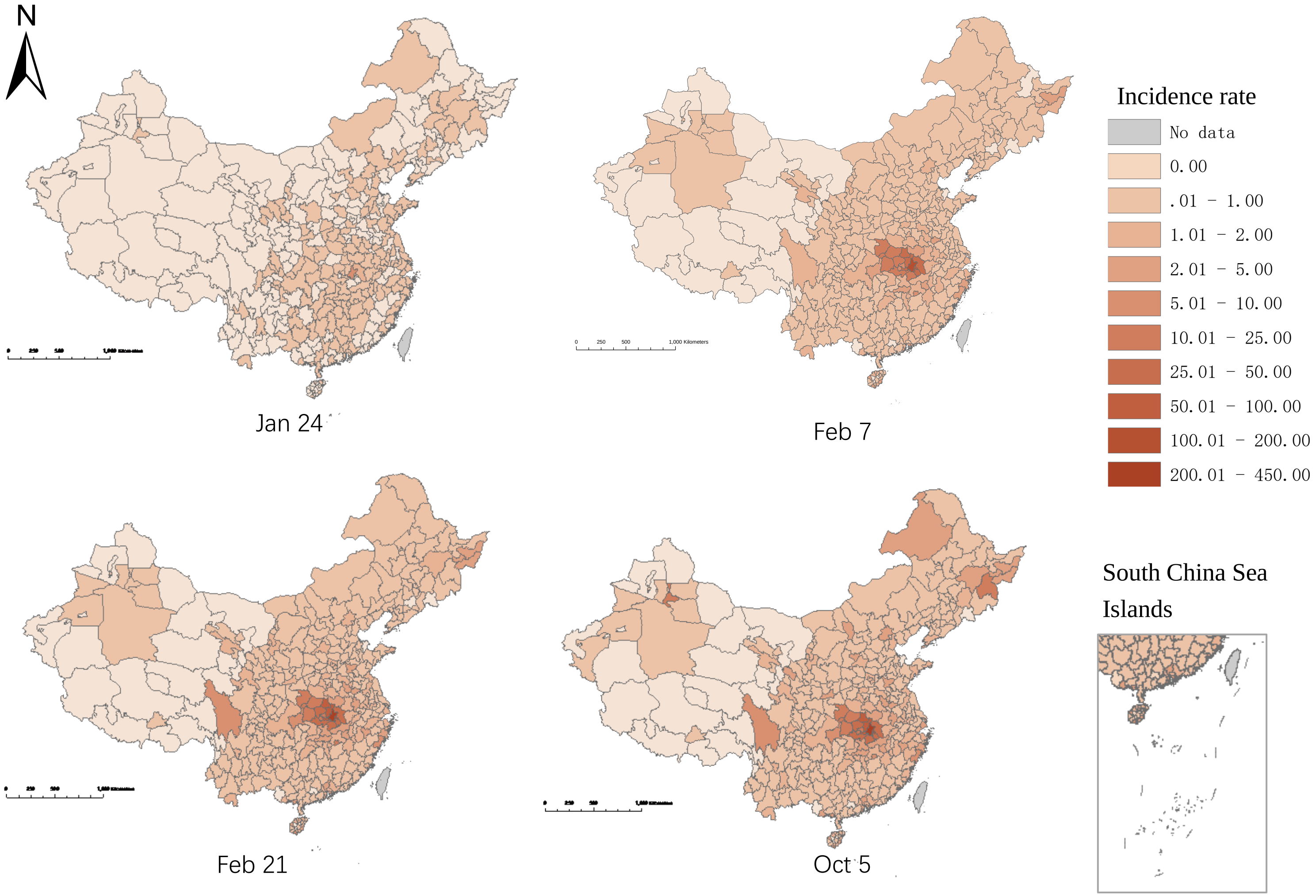

Figure 2 Spatio-temporal distribution of the incidence of COVID-19 in mainland China
